# Association of Remnant Cholesterol With Self‑Reported Trouble Sleeping and Mediation by Depression

**DOI:** 10.1002/brb3.71201

**Published:** 2026-01-07

**Authors:** Qichao Yang, Zhaoxiang Wang, Han Yan, Xuejing Shao

**Affiliations:** ^1^ Department of Endocrinology Affiliated Wujin Hospital of Jiangsu University, Wujin Clinical College of Xuzhou Medical University Changzhou Jiangsu China; ^2^ Department of Endocrinology Affiliated Kunshan Hospital of Jiangsu University Kunshan Jiangsu China

**Keywords:** depression, linear relationship, dyslipidemia, remnant cholesterol, trouble sleeping

## Abstract

**Purpose:**

Remnant cholesterol (RC) is an independent risk factor for cardiovascular diseases. The objective of this study is to assess the correlation between RC levels and trouble sleeping among U.S. adults.

**Methods:**

This study analyzed data from 14,617 adults enrolled in the National Health and Nutrition Examination Survey (NHANES), calculating RC by subtracting both high‐density and low‐density lipoprotein cholesterol (HDL‐c and LDL‐c) from total cholesterol (TC). Trouble sleeping was identified through self‐report history. The relationship between RC and trouble sleeping was explored through logistic regression, restricted cubic spline (RCS), and mediation analysis.

**Results:**

The prevalence of trouble sleeping escalates with increasing RC levels. Adjusting for potential confounders, higher RC levels were significantly associated with an increased risk of trouble sleeping [OR (95% CI): 1.30 (1.13–1.49), ***p*** < 0.001]. A linear relationship was identified through RCS analysis. Moreover, depression was found to partially mediate the relationship between RC and trouble sleeping.

**Conclusions:**

Trouble sleeping is associated with increased RC levels. The causal relationship requires additional investigation.

## Graphical abstract

Remnant cholesterol was positively and linearly associated with self‐reported trouble sleeping in U.S. adults, and depression partially mediated this relationship. Monitoring remnant cholesterol may help identify individuals at higher risk of sleep problems and related cardiometabolic consequences.

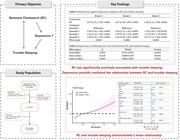

## 1 Introduction

As the pace of society continues to increase, sleep health has emerged as a significant concern for public health, with about one‐third of the population expressing dissatisfaction with their sleep (Barnes and Drake 2015; Ohayon 2002; National Institutes of Health 2005). Trouble sleeping, encompassing conditions like obstructive sleep apnea, issues related to sleep quality (including inadequate sleep, sleep duration, and insomnia), and a variety of other sleep issues, is progressively prevalent among adults in the United States (You et al. 2023; Sateia 2014). These sleep issues often accompany physical or mental health conditions, such as dyslipidemia, metabolic disorders, depression, and cardiovascular diseases (Chaput et al. 2023; Shan et al. 2015; Miller and Howarth 2023; Chunnan et al. 2022; Drager et al. 2010).

Recent studies suggest that remnant cholesterol (RC), the cholesterol component found in triglyceride (TG)‐rich lipoproteins (TRLs) like very‐low‐density lipoproteins, intermediate‐density lipoproteins, and chylomicron remnants, may be a crucial factor in the development of atherosclerotic cardiovascular disease (ASCVD) (Quispe et al. 2021; Jørgensen et al. 2013). Unlike low‐density lipoprotein cholesterol (LDL‐c), RC has been identified as having a more direct impact on cardiovascular health, marking it as a focal point in lipid management strategies (Quispe et al. 2021; Cao et al. 2023). Furthermore, emerging research points to RC as a promising marker for various metabolic disorders, including diabetes, obesity, metabolic syndrome, and non‐alcoholic fatty liver disease (NAFLD) (Hu et al. 2022; Tong et al. 2022; Zou et al. 2023; Huang et al. 2023).

It is well established that dyslipidemia and sleep disorders interact, yet most existing studies have relied on baseline lipid profiles, and it remains unclear whether trouble sleeping is associated with elevated RC levels and whether RC can effectively distinguish individuals prone to sleep problems. By employing the National Health and Nutrition Examination Survey (NHANES) database, this cross‐sectional investigation examines the relationship between RC and trouble sleeping among adults in the United States.

## 2 Materials and Methods

### 2.1 Study Population

**FIGURE 1:**
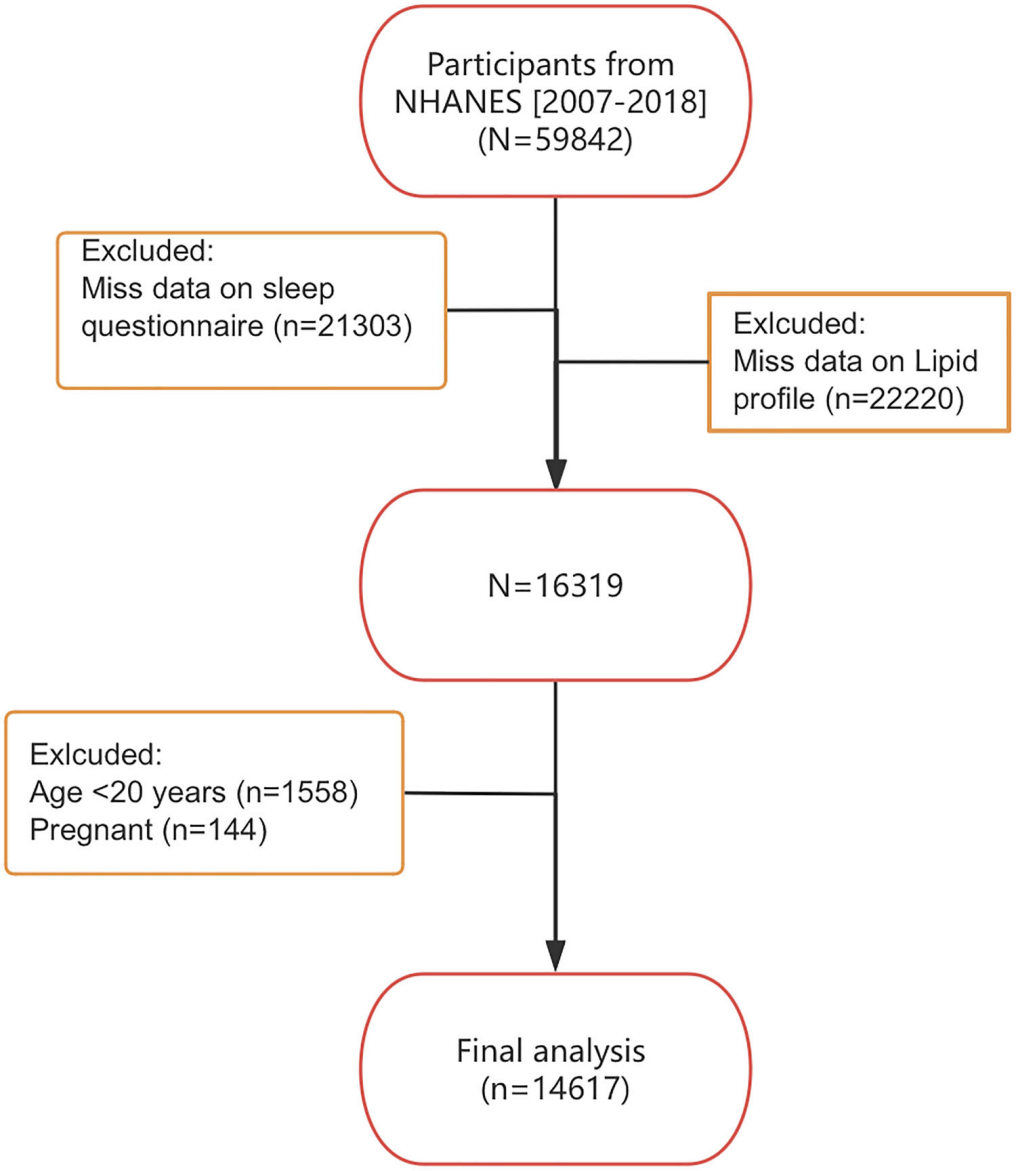
Flowchart of participant screening.

The NHANES, carried out by the National Center for Health Statistics (NCHS), is a periodic survey designed to evaluate the health and nutritional status of the United States population, employing a complex multistage stratified sampling approach (https://wwwn.cdc.gov/nchs/nhanes) (Ahluwalia et al. 2016; Paulose‐Ram et al. 2021). Written consent was obtained from all participants, and the NCHS Research Ethics Review Board approved the study (https://www.cdc.gov/nchs/nhanes/about/erb.html). Our research utilized data from six NHANES cycles, from 2007–2008 to 2017–2018, excluding those under 20 years of age, pregnant individuals, and participants lacking lipid profile and sleep questionnaire data (Figure 1). Finally, our study included 14,617 participants.

### 2.2 Measures of Exposure and Outcome

According to the guidelines on dyslipidemia by the European Atherosclerosis Society, RC is derived by deducting both high‐density lipoprotein cholesterol (HDL‐c, mmol/L) and LDL‐c (mmol/L) from the total cholesterol (TC, mmol/L) in the lipid profile (Mach et al. 2020). The history of self‐reported trouble sleeping was gathered via the question, “Have you ever told a doctor or other health professional that you have trouble sleeping?” with possible responses being “Yes,” “No,” “Refused,” and “Don't know.” Both “Refused” and “Don't know” responses were considered as missing values. The reliability of self‐reported trouble sleeping has been confirmed in previous studies and utilized in epidemiological research on sleep issues within the United States population (You et al. 2023; Chunnan et al. 2022; Di et al. 2022; Lei et al. 2023; Wang et al. 2024).

### 2.3 Covariates

Based on prior literature, our study accounted for various potential confounders, including demographic factors (age, gender, race, marital status), socioeconomic status (household income, education level), health behaviors (smoking status), and medical conditions (diabetes, hypertension, cardiovascular diseases, depression) (Wang et al. 2025; Wang et al. 2025; Wang and Shen 2023). It also considered physiological and biochemical measures such as body mass index (BMI), glycated hemoglobin (HbA1c), liver enzymes (alanine transaminase [ALT], aspartate transaminase [AST], and gamma‐glutamyl transferase [GGT]), triglycerides (TG), serum uric acid (SUA), serum creatinine (Scr), and the estimated glomerular filtration rate (eGFR). The eGFR was calculated via the Chronic Kidney Disease Epidemiology Collaboration (CKD‐EPI) formula based on age, gender, race, and Scr levels (Levey et al. 2009). Diabetes and hypertension diagnoses were self‐reported, while cardiovascular disease identification required reports of heart attacks, strokes, heart failure, coronary artery disease, or angina. Depression was assessed using the Patient Health Questionnaire (PHQ‐9), with scores of 10 or above indicating major depression (Negeri et al. 2021).

### 2.4 Statistical Analysis

Population‐weighted approaches were utilized for statistical analysis. Continuous variables were reported as mean values with standard errors (SE), while categorical data were shown as weighted percentages. Group differences were evaluated using weighted t‐tests for continuous variables and weighted chi‐square tests for categorical variables. To investigate the independent association between RC and trouble sleeping, three weighted logistic regression models were developed: Model 1 without any covariate adjustments, Model 2 adjusted for age, gender, and race, and Model 3 further adjusted for marital status, household income, education level, smoking status, diabetes, hypertension, cardiovascular diseases, BMI, HbA1c, ALT, AST, GGT, SUA, Scr, and eGFR. The Variance Inflation Factor (VIF) was used to evaluate collinearity. Restricted cubic spline (RCS) regression with four knots was applied, taking the median value as the reference to examine possible non‑linear relationships. Subgroup analyses were also performed. The potential mediation effect of depression on the RC‐trouble sleeping association was examined using the Bootstrap approach. Statistical analyses were carried out using Stata 18.0, R software, and EmpowerStats (http://www.empowerstats.com). The analysis adhered to a multi‐stage survey design with six sample weights according to the Centers for Disease Control and Prevention (CDC) guidelines, considering a two‐sided ***p***‐value < 0.05 as statistically significant.

## 3 Results

### 3.1 Baseline Characteristics of Study Population

This study analyzed a cohort of 14,617 participants (Table 1). Compared with participants without trouble sleeping, those reporting trouble sleeping were older, more often female, had lower household income, and were more likely to smoke (***p*** < 0.001). They also had a higher prevalence of hypertension, diabetes, cardiovascular diseases, and depression, as well as higher mean BMI, HbA1c, ALT, AST, TG, and TC, and lower eGFR (all ***p*** < 0.05). Importantly, RC levels were significantly higher in the trouble sleeping group than in those without trouble sleeping (0.64 ± 0.01 vs. 0.58 ± 0.01 mmol/L, ***p*** < 0.001). Participants were also divided into four groups based on RC levels using quartile distribution (Table 2). When compared to the group in the lowest RC quartile, the high RC quartile group was older, more often male and married, had lower annual household incomes, and demonstrated greater prevalence of smoking, diabetes, hypertension, cardiovascular diseases, and depression (***p*** < 0.01). Additionally, elevated levels of BMI, HBA1c, ALT, AST, GGT, TG, TC, LDL‐c, SUA, and Scr were observed, while education level above high school, eGFR, and HDL‐c levels were lower (***p*** < 0.001). Importantly, as RC levels increased, there was a corresponding rise in the prevalence of trouble sleeping (22.73% vs. 27.80% vs. 29.17% vs. 32.09%, ***p*** < 0.001).

**TABLE 1:** Baseline characteristics of participants by sleep status.

	Overall (*N* = 14,617)	Non‐trouble sleeping (N = 10,808)	Trouble sleeping (*N* = 3809)	*p*‐value
Age (years)	48.21 ± 0.26	46.83 ± 0.29	51.77 ± 0.34	< 0.001
Male gender, %	48.48	51.44	40.83	< 0.001
Race, %				< 0.001
Mexican American	8.55	9.87	5.16	
Non‐Hispanic Black	10.52	10.74	9.96	
Non‐Hispanic White	66.93	64.05	74.37	
Other Hispanic	6.03	6.62	4.51	
Other Races	7.96	8.72	5.99	
Married, %	55.46	56.72	52.22	< 0.001
Annual household income (under $20,000), %	14.15	13.13	16.76	< 0.001
Education level (above high school), %	60.68	60.44	61.31	0.516
Smokers, %	44.72	41.11	54.05	< 0.001
Diabetes, %	9.96	8.34	14.16	< 0.001
Hypertension, %	33.43	28.50	46.13	< 0.001
Cardiovascular diseases, %	9.32	7.26	14.62	< 0.001
Depression, %	7.77	3.96	17.39	< 0.001
BMI (kg/m^2^)	29.01 ± 0.10	28.57 ± 0.11	30.14 ± 0.17	< 0.001
HbA1c (%)	5.65 ± 0.01	5.61 ± 0.01	5.75 ± 0.02	< 0.001
ALT (U/L)	24.88 ± 0.18	24.66 ± 0.20	25.75 ± 0.36	0.005
AST (U/L)	24.97 ± 0.16	24.67±0.16	25.74±0.37	0.008
GGT (U/L)	27.43 ± 0.31	26.18 ± 0.37	30.67 ± 0.65	< 0.001
TG (mmol/L)	1.31 ± 0.01	1.27 ± 0.01	1.40 ± 0.02	< 0.001
TC (mmol/L)	4.95 ± 0.01	4.93 ± 0.02	4.99 ± 0.02	0.024
HDL‐c (mmol/L)	1.41 ± 0.01	1.41 ± 0.01	1.42 ± 0.01	0.266
LDL‐c (mmol/L)	2.94 ± 0.01	2.94 ± 0.01	2.93 ± 0.02	0.658
SUA (µmol/L)	325.94 ± 1.05	326.71 ± 1.26	323.95 ± 1.94	0.242
Scr (µmol/L)	77.69 ± 0.33	77.43 ± 0.32	78.37 ± 0.80	0.267
eGFR (ml/min/1.73 m^2^)	94.83 ± 0.35	96.30 ± 0.41	91.04 ± 0.49	< 0.001
RC (mmol/L)	0.60 ± 0.01	0.58 ± 0.01	0.64 ± 0.01	< 0.001

**Abbreviations**: ALT, alanine transaminase; AST, aspartate transaminase; BMI, body mass index; eGFR, estimated glomerular filtration rate; GGT, gamma‐glutamyl transferase; HbA1c, glycated hemoglobin; HDL‐c, high‐density lipoprotein cholesterol; LDL‐c, low‐density lipoprotein cholesterol; RC, remnant cholesterol; Scr, serum creatinine; SUA, serum uric acid; TC, total cholesterol; TG, triglyceride.

**TABLE 2:** Baseline characteristics of participants grouped by RC quartiles.

	Quartile 1	Quartile 2	Quartile 3	Quartile 4	*p*‐value
Age (years)	43.93 ± 0.47	48.37 ± 0.44	49.76 ± 0.39	50.91 ± 0.35	< 0.001
Male gender, %	42.25	46.39	49.85	55.60	< 0.001
Race, %					< 0.001
Mexican American	6.57	7.17	10.02	10.50	
Non‐Hispanic Black	17.18	11.84	7.81	5.06	
Non‐Hispanic White	63.06	66.87	67.84	70.08	
Other Hispanic	5.36	6.00	6.28	6.49	
Other Races	7.82	8.12	8.04	7.87	
Married, %	52.44	54.17	56.93	58.40	0.001
Annual household income (under $20,000), %	12.04	14.16	15.16	15.28	0.002
Education level (above high school), %	67.49	61.25	59.19	54.59	< 0.001
Smokers, %	37.57	43.01	45.84	52.69	< 0.001
Diabetes, %	5.09	7.97	10.64	16.30	< 0.001
Hypertension, %	22.11	31.68	37.04	43.22	< 0.001
Cardiovascular diseases, %	6.08	8.94	10.34	12.00	< 0.001
Depression, %	6.36	6.88	7.03	10.81	< 0.001
BMI (kg/m^2^)	26.64 ± 0.18	28.32 ± 0.16	29.89 ± 0.16	31.25 ± 0.17	< 0.001
HbA1c (%)	5.43 ± 0.01	5.57 ± 0.02	5.67 ± 0.02	5.93 ± 0.03	< 0.001
ALT (U/L)	21.49 ± 0.39	23.65 ± 0.32	25.39 ± 0.27	29.11 ± 0.35	< 0.001
AST (U/L)	24.18 ± 0.33	24.76 ±0.33	24.68 ± 0.22	26.29 ± 0.34	< 0.001
GGT (U/L)	21.08 ± 0.49	24.44 ± 0.58	28.39 ± 0.75	36.02 ± 0.74	< 0.001
TG (mmol/L)	0.59 ± 0.00	0.95 ± 0.00	1.36 ± 0.00	2.35 ±0.02	< 0.001
TC (mmol/L)	4.51 ± 0.02	4.84 ± 0.02	5.06±0.02	5.40 ± 0.02	< 0.001
HDL‐c (mmol/L)	1.65 ± 0.01	1.48 ± 0.01	1.35 ± 0.01	1.15 ± 0.01	< 0.001
LDL‐c (mmol/L)	2.58 ± 0.02	2.92 ± 0.02	3.09 ± 0.02	3.17 ± 0.02	< 0.001
SUA (µmol/L)	295.39 ± 1.67	318.43 ± 1.79	335.11 ± 2.12	355.72 ± 1.78	< 0.001
Scr (µmol/L)	74.24 ± 0.36	78.10 ± 0.76	78.71 ± 0.60	79.83 ± 0.72	< 0.001
eGFR (ml/min/1.73 m^2^)	100.24 ± 0.53	94.36 ± 0.60	92.84 ± 0.52	91.73 ± 0.49	< 0.001
Trouble sleeping, %	22.73	27.80	29.17	32.09	< 0.001

### 3.2 The Association Between RC Levels and Trouble Sleeping

**FIGURE 2:**
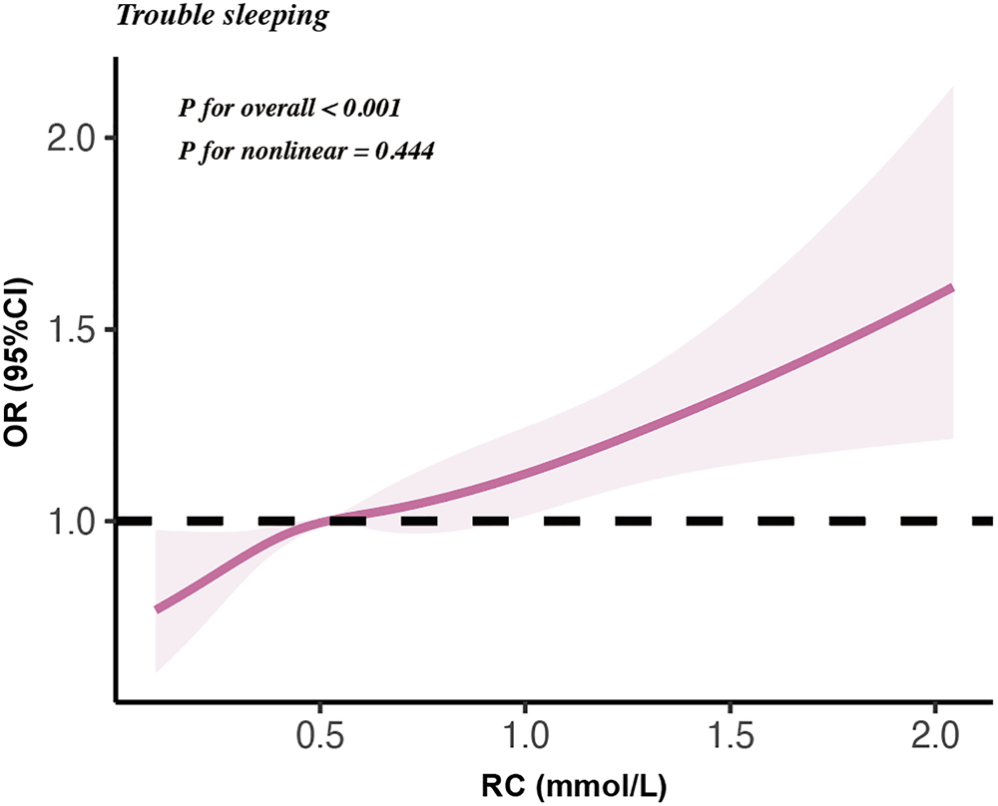
Results of RCS analysis.

Table 3 presents a positive association between RC and trouble sleeping, stable across Models 1, 2, and 3. In Model 3, after fully adjusting for confounding factors, a one‐unit increase in RC levels is associated with a 30% higher risk of trouble sleeping [OR (95% CI): 1.30 (1.13‐1.49), ***p*** < 0.001]. Upon dividing RC levels into quartiles, the relationship remained statistically significant (***p*** < 0.001). Individuals in the top quartile for RC levels had a 27% higher likelihood of experiencing trouble sleeping than those in the bottom quartile [OR (95% CI): 1.27 (1.11–1.46), ***p*** < 0.001]. Further RCS analysis between RC levels and trouble sleeping revealed a linear relationship (Figure 2).

**TABLE 3:** Results from logistic regression analysis on RC and trouble sleeping.

	OR (95%CI) ***p***‐value		
	Model 1	Model 2	Model 3
Continuous			
RC	1.57 (1.41, 1.74) < 0.001	1.67 (1.49, 1.87) < 0.001	1.30 (1.13, 1.49) < 0.001
Categories			
Quantile 1	Reference	Reference	Reference
Quantile 2	1.29 (1.16, 1.44) <0.001	1.26 (1.13, 1.41) <0.001	1.16 (1.03, 1.32) 0.018
Quantile 3	1.36 (1.22, 1.52) <0.001	1.35 (1.21, 1.51) <0.001	1.17 (1.03, 1.33) 0.016
Quantile 4	1.58 (1.42, 1.76) <0.001	1.62 (1.45, 1.81) <0.001	1.27 (1.11, 1.46) <0.001
***P*** for trend	< 0.001	< 0.001	< 0.001

OR: odds ratio.

95% CI: 95% confidence interval.

Model 1: no covariates adjusted.

Model 2: adjusted for age, gender, and race.

Model 3: adjusted for age, gender, and race, marital status, household income, education level, smoking status, diabetes, hypertension, cardiovascular diseases, BMI, HbA1c, ALT, AST, GGT, SUA, Scr, and eGFR.

### 3.3 Subgroup Analysis

**FIGURE 3:**
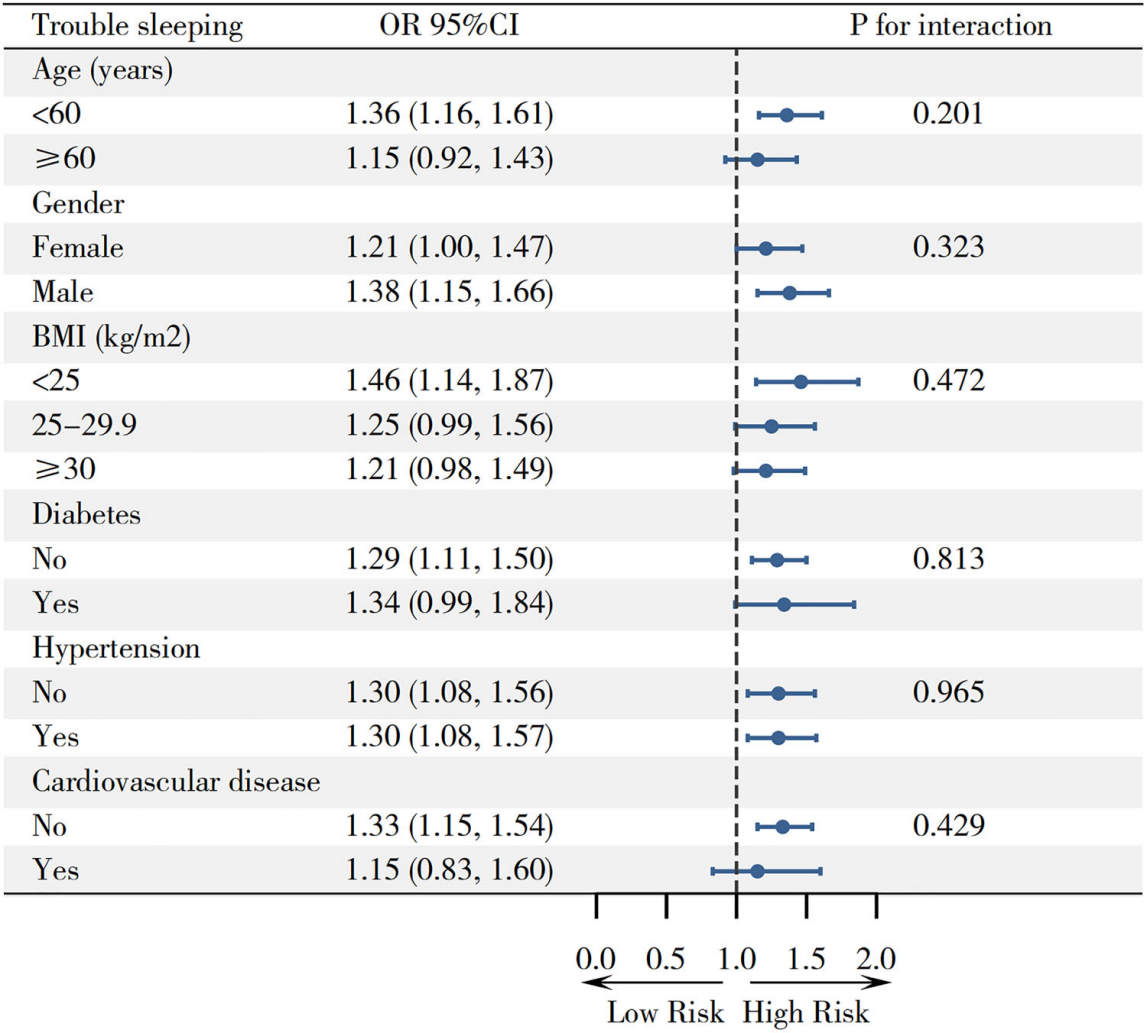
Results of subgroup analysis.

We further examined whether the association between RC and trouble sleeping differed across clinically relevant subgroups (Figure 3). By age, the fully adjusted ORs for trouble sleeping per unit increase in RC were 1.36 (95% CI: 1.16–1.61) in participants aged <60 years and 1.15 (95% CI: 0.92–1.43) in those aged ≥60 years (***P*** for interaction = 0.201). The association was present in both females and males, with ORs of 1.21 (1.00–1.47) and 1.38 (1.15–1.66), respectively (***P*** for interaction = 0.323). Across BMI categories, the ORs were 1.46 (1.14–1.87) for BMI < 25 kg/m^2^, 1.25 (0.99–1.56) for BMI 25–29.9 kg/m^2^, and 1.21 (0.98–1.49) for BMI ≥30 kg/m^2^ (***P*** for interaction = 0.472). Similar patterns were observed when stratified by diabetes (no vs. yes: 1.29 [1.11–1.50] vs. 1.34 [0.99–1.84]; ***P*** for interaction = 0.813), hypertension (no vs. yes: 1.30 [1.08–1.56] vs. 1.30 [1.08–1.57]; ***P*** for interaction = 0.965), and cardiovascular disease (no vs. yes: 1.33 [1.15–1.54] vs. 1.15 [0.83–1.60]; ***P*** for interaction = 0.429).

### 3.4 Mediator Analysis

Considering depression as a significant factor related to trouble sleeping, we further explored the mediation impact of depression on the relationship between RC levels and trouble sleeping (Table 4) (Chunnan et al. 2022). After adjusting for covariates, the results highlight a significant mediation role of depression in connecting RC levels with trouble sleeping (***p*** < 0.001). Both direct and indirect effects were found to be significant (***p*** < 0.001). This mediation effect explains 21.74% (0.005/0.023) of the total variance (Supplementary Table S1).

**TABLE 4:** Depression's mediating role in the link between RC and trouble sleeping.

Effect types	*β*	95% CI	*p*‐value
Total effect	0.023	0.012–0.031	< 0.001
Direct effect	0.018	0.008–0.027	< 0.001
Indirect effect	0.005	0.002–0.007	< 0.001

adjusted for age, gender, and race, marital status, household income, education level, smoking status, diabetes, hypertension, cardiovascular diseases, BMI, HbA1c, ALT, AST, GGT, SUA, Scr, and eGFR.

### 3.5 Sensitivity Analysis

The robustness of the association between RC and trouble sleeping was further evaluated using sensitivity analyses. First, we re‑estimated the models after excluding participants using lipid‑lowering medications (Supplementary Table S2). Second, we repeated the analyses in a subsample restricted to individuals with RC levels in the normal range (<0.62 mmol/L), as defined by the 2019 ESC/EAS Guidelines, to assess whether the association held under typical physiological conditions (Supplementary Table S3) (Mach et al. 2020). In both analyses, the direction and magnitude of the association were consistent with the primary results.

## 4 Discussion

This cross‐sectional analysis revealed a significant positive association between RC levels and the risk of trouble sleeping among the U.S. adult population. Additionally, depression was found to partially mediate this relationship.

While LDL‐c traditionally stands as the cornerstone in dyslipidemia treatment, the advent and common use of statins have shifted focus toward TRLs, emblematic of statin‐refractory dyslipidemia (Wadström et al. 2023). An extensive cohort study over 18 years revealed that increased RC levels contribute to the risk of atherosclerotic cardiovascular disease (ASCVD) beyond what is accounted for by conventional risk factors such as LDL‐c and apolipoprotein B (ApoB) (Quispe et al. 2021). Consequently, there is a growing viewpoint that RC might play a more significant role in cardiovascular disease development than LDL‐c (Castañer et al. 2020). Current research suggests that there is a bidirectional association between trouble sleeping and dyslipidemia and cardiovascular diseases (Sánchez‐de‐la‐Torre and Barbé 2022). Poor sleep quality contributes substantially to the development and exacerbation of cardiovascular disease (Javaheri et al. 2017; Fan et al. 2020). On the other hand, heart failure and coronary artery disease can trigger symptoms like chest pain, difficulty breathing, and frequent nighttime urination, which disrupt sleep. Additionally, many cardiovascular conditions are linked to autonomic nervous system imbalances, altering heart rate, blood pressure, and breathing patterns during sleep (Freeman et al. 2006). Moreover, medications prescribed for these conditions, including diuretics and beta‐blockers, can lead to side effects such as nocturia and nightmares, further impacting sleep (Tabara and Chin 2021; Pope 2021). In patients with diagnosed trouble sleeping, monitoring lipid profiles, particularly RC levels, could be crucial in preventing ASCVD. Conversely, for patients with elevated RC levels, screening for trouble sleeping could be beneficial, as early detection and management of trouble sleeping.

The pathophysiological mechanisms underlying the relationship between RC and trouble sleeping are multifaceted. Elevated levels of RC can lead to endothelial dysfunction, inflammation, and atherosclerosis, which in turn may contribute to the development of trouble sleeping through various pathways. For instance, atherosclerosis can lead to reduced oxygenation during sleep, exacerbating sleep‐disordered breathing (Tarbell et al. 2020; Batty et al. 2022). Conversely, trouble sleeping, particularly obstructive sleep apnea (OSA), can induce metabolic dysregulation, promoting dyslipidemia. Evidence indicates that during fasting, OSA and intermittent hypoxia (IH) enhance the flow of lipids from adipose tissue to the liver by increasing the activity of sterol regulatory element‐binding protein‐1 and stearoyl‐CoA desaturase‐1, evaluating the production of cholesterol esters and triglycerides (Barros and García‐Río 2019). In the postprandial phase, the clearance of lipoproteins slows down, attributed to reduced activity of lipoprotein lipase (Barros and García‐Río 2019). The stress and inflammation resulting from poor sleep quality can also exacerbate lipid abnormalities, creating a vicious cycle. Trouble sleeping correlates with extensive metabolic disorders, including glucose intolerance, insulin resistance, susceptibility to type 2 diabetes, and metabolic syndrome in adults (Wei et al. 2022). RC can induce endoplasmic reticulum stress and mitochondrial dysfunction in pancreatic β‐cells, leading to increased reactive oxygen species (ROS) production and β‐cell damage (Hu et al. 2022). Unlike LDL‐C, RC's unique pro‐inflammatory properties may exacerbate insulin resistance and systemic inflammation, significantly contributing to metabolic dysregulation (Hu et al. 2022). Additionally, sleep issues are also associated with mental health (Barsha and Hossain 2020). Based on the analysis of mediating effects, we found that depression mediates the relationship between RC and trouble sleeping. In our study, we observed that as RC levels increase, the prevalence of depression also rises. Previous research has also confirmed that RC is associated with an increased risk of depression (Wang and Shen 2023). Nevertheless, the mediated proportion of 21.74% indicates that other pathways, such as RC‑related inflammation and metabolic comorbidities, may also contribute and warrant further investigation. In terms of mechanism, RC participates in regulating the activation of the hypothalamic‐pituitary‐adrenal (HPA) axis, cytokine secretion of neurons, and dysfunction of cerebral microvascular function, all of which are involved in the pathogenesis of depression (Wang and Shen 2023). In age‐stratified analyses, the association between RC and trouble sleeping was stronger among participants aged <60 years than among those ≥60 years, although the *p*‐value for interaction was not statistically significant. This pattern may reflect that adults <60 years are more often in earlier or subclinical phases of cardiometabolic disease, in which RC‐related endothelial dysfunction, low‐grade inflammation, and autonomic dysregulation exert a relatively larger influence on sleep regulation before multiple comorbidities accumulate. By contrast, in older adults, a greater burden of chronic conditions and polypharmacy may attenuate or obscure the independent impact of RC on trouble sleeping.

This study, utilizing sophisticated sampling weights, represents the demographic distribution of the United States. However, there are also some limitations in our study. First, the cross‐sectional design precludes causal inference between RC and trouble sleeping and limits the interpretability of the mediation analysis. In this context, depression can only be regarded as an associative statistical mediator rather than a causal mediator, and the mediation findings should be interpreted with caution. Second, despite adjustment for many confounders, some important factors that directly affect sleep—such as metabolic syndrome, NAFLD, psychological stress, and major life events—were not available. Finally, given that participants in our study are predominantly U.S. adults, extending our conclusions to a wider population should be done with caution.

## 5 Conclusion

This national cross‐sectional study provides evidence of a positive correlation between RC and trouble sleeping. Monitoring RC levels in patients with trouble sleeping might be instrumental in the prevention of cardiovascular diseases. Moreover, the potential mediation by depression could be an important contributing factor to this relationship.

## Author Contributions

**Qichao Yang**: conceptualization and writing – original draft. **Zhaoxiang Wang**: writing – original draft. **Han Yan**: formal analysis and writing – original draft. **Xuejing Shao**: supervision, funding acquisition, writing – review and editing. Qichao Yang and Zhaoxiang Wang contributed equally to this work.

## Funding

This study was supported by the Young Talent Development Plan of Changzhou Health Commission (CZQM2022029).

## Ethics Statement

This study involving human participants were reviewed and approved by the Ethics Review Board of the National Center for Health Statistics (https://www.cdc.gov/nchs/nhanes/about/erb.html).

## Consent

The patients/participants provided their written informed consent to participate in this study.

## Conflicts of Interest

The authors declare no conflicts of interest.

## Supporting information




**Table S1** Association of RC with depression and of depression with trouble sleeping.
**Table S2** Sensitivity analysis of the association between RC and trouble sleeping after excluding participants using lipid‑lowering medications.
**Table S3** Sensitivity analysis of the association between RC (<0.62 mmol/L) and trouble sleeping.

## Data Availability

The data is sourced from the NHANES database, which is a publicly accessible and free resource (https://wwwn.cdc.gov/nchs/nhanes).
