# Supplementary material for: Association of Remnant Cholesterol With Self‑Reported Trouble Sleeping and Mediation by Depression
Source: Brain Behav. 2026 Jan 7;16(1):e71201. doi: 10.1002/brb3.71201 (PMC12778401; doi:10.1002/brb3.71201)
Supplement: Supplementary file 1 — Table S1 Association of RC with depression and of depression with trouble sleeping. Table S2 Sensitivity analysis of the association between RC and trouble sleeping after excluding participants using lipid‑lowering medications. Table S3 Sensitivity analysis of the association between RC (<0.62 mmol/L) and trouble sleeping. [file BRB3-16-e71201-s001.docx]

| **Table S1** Association of RC with depression and of depression with trouble sleeping. | | | |
| --- | --- | --- | --- |
|  | OR (95%CI) ***P*** value | | |
|  | Model 1 | Model 2 | Model 3 |
| RC and depression | | | |
| Continuous | | | |
| RC | 1.85 (1.58, 2.17) <0.001 | 2.15 (1.83, 2.54) <0.001 | 1.62 (1.33, 1.96) <0.001 |
| Categories | | | |
| Quantile 1 | Reference | Reference | Reference |
| Quantile 2 | 1.15 (0.96, 1.38) 0.140 | 1.21 (1.01, 1.46) 0.043 | 1.02 (0.84, 1.25) 0.836 |
| Quantile 3 | 1.18 (0.98, 1.41) 0.079 | 1.28 (1.06, 1.54) 0.009 | 1.07 (0.88, 1.32) 0.488 |
| Quantile 4 | 1.80 (1.52, 2.13) <0.001 | 2.05 (1.72, 2.44) <0.001 | 1.48 (1.21, 1.81) <0.001 |
| ***P*** for trend | <0.001 | <0.001 | <0.001 |
| Depression and trouble sleeping |  |  |  |
| Depression |  |  |  |
| No | Reference | Reference | Reference |
| Yes | 4.87 (4.30, 5.51) <0.001 | 4.84 (4.26, 5.49) <0.001 | 4.09 (3.56, 4.70) <0.001 |
| OR: odds ratio.  95% CI: 95% confidence interval.  Model 1: no covariates adjusted.  Model 2: adjusted for age, gender, and race.  Model 3: adjusted for age, gender, and race, marital status, household income, education level, smoking status, diabetes, hypertension, cardiovascular diseases, BMI, HbA1c, ALT, AST, GGT, SUA, Scr, and eGFR. | | | |

| **Table S2** Sensitivity analysis of the association between RC and trouble sleeping after excluding participants using lipid‑lowering medications. | | | |
| --- | --- | --- | --- |
|  | OR (95%CI) ***P*** value | | |
|  | Model 1 | Model 2 | Model 3 |
| Continuous | | | |
| RC | 1.46 (1.29, 1.65) <0.001 | 1.60 (1.40, 1.82) <0.001 | 1.29 (1.10, 1.51) 0.002 |
| Categories | | | |
| Quantile 1 | Reference | Reference | Reference |
| Quantile 2 | 1.28 (1.13, 1.45) <0.001 | 1.26 (1.11, 1.44) <0.001 | 1.17 (1.01, 1.36) 0.039 |
| Quantile 3 | 1.33 (1.17, 1.51) <0.001 | 1.35 (1.18, 1.54) <0.001 | 1.18 (1.01, 1.37) 0.033 |
| Quantile 4 | 1.50 (1.33, 1.70) <0.001 | 1.58 (1.39, 1.81) <0.001 | 1.29 (1.10, 1.51) 0.001 |
| ***P*** for trend | <0.001 | <0.001 | 0.003 |
| OR: odds ratio.  95% CI: 95% confidence interval.  Model 1: no covariates adjusted.  Model 2: adjusted for age, gender, and race.  Model 3: adjusted for age, gender, and race, marital status, household income, education level, smoking status, diabetes, hypertension, cardiovascular diseases, BMI, HbA1c, ALT, AST, GGT, SUA, Scr, and eGFR. | | | |

| **Table S3** Sensitivity analysis of the association between RC (<0.62 mmol/L) and trouble sleeping. | | | |
| --- | --- | --- | --- |
|  | OR (95%CI) ***P*** value | | |
|  | Model 1 | Model 2 | Model 3 |
| Continuous | | | |
| RC | 2.89 (1.95, 4.27) <0.001 | 2.61 (1.73, 3.95) <0.001 | 1.64 (1.04, 2.58) 0.032 |
| Categories | | | |
| Quantile 1 | Reference | Reference | Reference |
| Quantile 2 | 1.23 (1.07, 1.41) 0.004 | 1.17 (1.02, 1.35) 0.029 | 1.11 (0.95, 1.29) 0.193 |
| Quantile 3 | 1.37 (1.19, 1.57) <0.001 | 1.31 (1.14, 1.52) <0.001 | 1.17 (1.00, 1.36) 0.048 |
| Quantile 4 | 1.38 (1.20, 1.58) <0.001 | 1.33 (1.15, 1.53) <0.001 | 1.15 (0.99, 1.35) 0.075 |
| ***P*** for trend | <0.001 | <0.001 | 0.065 |
| OR: odds ratio.  95% CI: 95% confidence interval.  Model 1: no covariates adjusted.  Model 2: adjusted for age, gender, and race.  Model 3: adjusted for age, gender, and race, marital status, household income, education level, smoking status, diabetes, hypertension, cardiovascular diseases, BMI, HbA1c, ALT, AST, GGT, SUA, Scr, and eGFR. | | | |
